# Neuregulin-1 promotes early regenerative and autophagic responses after ischemic stroke via spatial proteomics

**DOI:** 10.3389/fncel.2026.1739619

**Published:** 2026-06-02

**Authors:** Jessica M. Noll, Christopher McGinley, Catherine J. Augello, Esra Kürüm, Liuliu Pan, Anna Pavenko, Andy Nam, Byron D. Ford, Gregory D. Ford

**Affiliations:** 1 Division of Biomedical Sciences, University of California-Riverside School of Medicine, Riverside, CA, United States; 2 Department of Statistics, University of California, Riverside, Riverside, CA, United States; 3 Department of Anatomy, Howard University College of Medicine, Washington, DC, United States; 4 Nanostring Technologies, Seattle, WA, United States; 5 Department of Bioengineering, University of California, Riverside, Riverside, CA, United States

**Keywords:** autophagy, digital spatial profiling, ischemic stroke, mitophagy, neuregulin-1, neuroregeneration

## Abstract

Ischemic stroke remains a leading cause of death and long-term disability, yet effective treatments that promote recovery beyond the acute phase are lacking. Neuregulin-1 (NRG-1) has shown potent neuroprotective and anti-inflammatory properties in preclinical stroke models, with evidence of enhanced neuronal regeneration when administered after injury. To investigate the spatial mechanisms underlying its neuroregenerative therapeutic effects, we examined brain proteomic responses to post-ischemic NRG-1 treatment in mice using NanoString Digital Spatial Profiling (DSP). Adult C57BL/6 mice were subjected to photothrombotic middle cerebral artery occlusion (MCAO) and treated with NRG-1β (5 μg/kg/day) or vehicle at 24- and 48-h post-stroke. Brains were collected at 3 days post-ischemia for spatial proteomic analysis of 68 neural proteins across the ischemic core, peri-infarct tissue, and peri-infarct normal tissue (PiNT). While NRG-1 did not significantly alter overall neuronal death, it markedly reshaped the neuroregenerative milieu, upregulating myelin basic protein (MBP) and synaptophysin and attenuating inflammatory mediators (SPP1, P2RX7, and CD39). NRG-1 also enhanced expression of autophagy and mitophagy markers (ULK1, LC3B, ATG5, PINK1, and Park7), suggesting restoration of cellular clearance and mitochondrial quality control. Pathway and network analyses revealed activation of neuroregeneration, autophagy, and lysosomal biogenesis pathways, while suppressing neuroinflammatory signaling. These findings demonstrate that delayed NRG-1 therapy, even when initiated 24 h after stroke, induces early molecular programs that prime an anti-inflammatory and neuroregenerative environment. The results support further development of NRG-1 as a clinically translatable, multimodal therapy for extending the post-stroke treatment window and promoting functional recovery.

## Introduction

Stroke remains a leading cause of death and disability worldwide, yet no therapies are available to facilitate neuroprotection or post-stroke regeneration (Benjamin, 2017; Bosetti et al., 2017; Marín-Medina et al., 2024; Noll et al., 2024). Tissue plasminogen activator (tPA) is the only FDA-approved acute treatment, but its narrow therapeutic window and risk of hemorrhage limit applicability. Beyond neuroprotection, effective strategies are needed to enhance endogenous repair processes such as synaptic plasticity and remyelination. Despite promising preclinical results, most neuroprotective and neurorestorative agents have failed in clinical trials, underscoring a critical unmet need for regenerative therapies.

Brain ischemia is characterized by early onset neuronal death beginning within minutes following stroke. This initial area of brain injury (the infarct core) is characterized by low cerebral blood flow, energy failure, excitotoxicity, and edema (for reviews Barone and Feuerstein, 1999; Dirnagl et al., 1999). The resulting ischemic brain injury is also accompanied by increased synthesis of inflammatory molecules and oxidative stress in neurons, glia, and in the cerebral vasculature, which endangers brain cells in a larger, surrounding area of brain tissue for which the blood supply is compromised but not completely interrupted (del Zoppo et al., 2000; Iadecola and Alexander, 2001; Iadecola and Anrather, 2011; Stoll et al., 1998). This population of surviving, yet vulnerable cells make up the ischemic penumbra, which surrounds the necrotic core of the infarct (Dirnagl et al., 1999; Touzani et al., 2001). The expansion of the infarct core is a time-dependent situation where damage to the adjacent viable tissue continues to progress into the penumbra. After ischemic damage is complete, the remaining, bordering region of reversibly damaged cells in an area of decreased neuronal density is the peri-infarct tissue (Lipton, 1999). The peri-infarct tissue later becomes an important target for neurorepair with a growth permissive environment allowing for induction of axonal sprouting, new neuronal connections, and survival and maturation of new neurons (Carmichael, 2006).

Neuronal recovery requires the activation of long-term regenerative processes, including cellular debris removal/autophagy, synaptic plasticity and remyelination (Carmichael, 2006; Hermann and Chopp, 2012; Marín-Medina et al., 2024). Following ischemic injury, the adult brain exhibits limited but quantifiable endogenous repair mechanisms. Ischemic stroke profoundly disrupts endogenous synaptic plasticity and promotes demyelination, leading to long-term deficits in neural connectivity and repair. These molecular alterations correspond to structural synaptic loss and impaired neuronal communication within both the ischemic core and peri-infarct regions. The result is a collapse of local plasticity and diminished capacity for axonal sprouting and circuit reorganization, which are critical for post-stroke recovery. Stimulation of synaptic plasticity and axonal sprouting can contribute to neuronal circuit restructuring. In parallel, demyelination occurs due to ischemia-induced oligodendrocyte death and white matter degradation. Myelin basic protein (MBP), a marker of oligodendrocyte integrity, is substantially decreased after stroke, reflecting myelin breakdown and impaired axonal conduction. The loss of myelin not only disrupts signal transmission but also compromises axonal survival. Although ischemia activates some regenerative responses, such as the proliferation of oligodendrocyte precursor cells and limited remyelination in peri-infarct areas, these processes are often insufficient to restore full function without therapeutic intervention.

Autophagy is an essential cellular process that maintains homeostasis by degrading and recycling damaged organelles, misfolded proteins, and other intracellular debris through lysosomal pathways (Klionsky et al., 2016). Following ischemic stroke, cells experience severe metabolic and oxidative stress that disrupts energy balance and accelerates the accumulation of dysfunctional proteins and mitochondria. Autophagic flux is transiently upregulated during ischemia as an adaptive mechanism to clear toxic aggregates. However, dysregulated or excessive autophagy during reperfusion can exacerbate neuronal injury. Balanced autophagic activity is therefore critical for maintaining neuronal viability and facilitating tissue recovery. Through these clearance pathways, autophagy promotes cellular survival, restores energy homeostasis, and limits neuroinflammation after stroke.

Despite these intrinsic responses, spontaneous recovery is generally incomplete, underscoring the need for therapeutic interventions that maintain and/or amplify regenerative processes. Several potential neuroprotective agents, including glutamate antagonists, anti-inflammatory compounds, free radical scavenging agents and hematopoietic growth factors were shown to be effective in preclinical animal models of ischemia, but failed to demonstrate efficacy in clinical trials with human patients following stroke (O'Collins et al., 2006). Clinical trials for post-injury stroke repair have shown limited success as well. No compound is yet approved specifically to induce neuronal repair after stroke (Marín-Medina et al., 2024). Therefore, the identification of therapeutic agents that limit brain damage or enhance recovery of motor function through neuroprotective and neurorestorative mechanisms is needed.

Among candidate molecules, neuregulin-1 (NRG-1) has emerged as a potent modulator of both tissue protection and repair. NRG-1 binds to ErbB receptor tyrosine kinases, activating PI3K/Akt and MAPK/ERK signaling pathways that support neuronal survival, glial proliferation, and vascular remodeling (Cespedes et al., 2018; Falls, 2003; Noll et al., 2024). Exogenous NRG-1 administration reduces infarct size, attenuates apoptosis, and modulates inflammatory responses in animal models. We and others have previously demonstrated that exogenous NRG-1 treatment significantly decreases acute neuronal death and neuroinflammation after ischemic damage with a therapeutic window of >12 h (Guan et al., 2015; Li et al., 2007c, 2008; Noll et al., 2019; Shyu et al., 2004; Xu et al., 2004, 2006). Beyond neuroprotection, NRG-1 enhances regenerative responses, including neural progenitor cell proliferation, oligodendrocyte maturation and remyelination, and angiogenesis in peri-infarct regions (Li et al., 2007a). Glial growth factor 2 (GGF2), a soluble isoform of NRG-1β, has demonstrated a direct neuroregenerative effect with increased neuronal sprouting, synapse formation markers, and functional recovery 3 weeks after ischemia (Iaci et al., 2010, 2016). Clinical development of GGF2 has advanced into phase 1 and 2 trials for heart failure, providing important translational insight into safety and systemic tolerability (Lenihan et al., 2016; Parry et al., 2017).

Overall, ischemic brain injury triggers synaptic loss, impaired plasticity, and demyelination, creating a hostile environment for neural regeneration. Treatments that restore synaptic proteins and promote remyelination through oligodendrocyte support, such as NRG-1 signaling, can counteract these effects, reestablishing connectivity and facilitating functional recovery. Here, we used NanoString Digital Spatial Profiling (DSP) to examine how delayed NRG-1 treatment alters neuroregenerative spatial proteomic profiles relative to the ischemic damage. Spatial profiling represents a revolutionary advancement in molecular biology that integrates multiplex molecular data with spatial context, enabling preservation of the native tissue architecture (Hernandez et al., 2022; Merritt et al., 2020). We previously showed using DSP that cerebral ischemia induced a unique spatial profile relating to increased neuronal death, stimulation of pro-inflammatory responses, and dysregulation of autophagic pathways (Noll et al., 2022). Our current data showed that delayed NRG-1 treatment induced spatial proteomic profiles after stroke characterized by reducing inflammation, increased expression of autophagy- and mitophagy-related pathways, and enhancing neuronal plasticity to enhance the priming of a neuroregenerative niche.

## Materials and methods

### Animals

All animals used in these studies were treated humanely and with regard to alleviation of suffering and pain, and all protocols involving animals were approved by the IACUC of University of California-Riverside prior to the initiation of experimentation (protocol # AUP 20190021). Male and female C57BL6 mice (8–10 weeks old) were purchased from Jackson Laboratories (Cat# 000664, Bar Harbor, Maine) and housed with a 12-h daily light/dark cycle. Food and water were provided *ad libitum*. All surgical procedures were performed by sterile/aseptic techniques in accordance with institutional guidelines. The study was carried out in compliance with the ARRIVE guidelines.

### Photothrombotic middle cerebral occlusion

Animals were randomized using a random number table and subjected to left photothrombotic MCA occlusion (MCAO) as we previously described (Noll et al., 2022). Mice were anesthetized with 2% isoflurane and circulating air (N_2_O:O_2_ at approximately 2:1) and maintained anesthetized during the procedure *via* a modified gas tubing nose connection on a stereotaxic instrument. Eye lubricant was applied to protect the eyes and body temperature was maintained *via* a heating pad placed underneath the mice during surgery at 37°C. Rose Bengal (10 mg/mL; Cat# 330000, Sigma, Burlington, MA) was injected intraperitoneally (i.p.) at 10 mL/g and allowed to incubate for 8 min. The animal was stabilized in the stereotaxic instrument, and the scalp hair was removed. The scalp was disinfected with iodine and ethanol, then followed by a midline skin incision to expose the skull above the left sensorimotor cortex. A 2 mm diameter focal green laser (520 nm; Cat# LP520-MF100 ThorLabs Inc, Newton, NJ) was directed at 2 mm lateral left and 0.6 mm posterior of bregma. Laser irradiation occurred for 20 min at 10 mW. After irradiation, the midline incision was sealed with Vetbond glue (Cat# NC0398332, Fisher Scientific, Hampton, NH) followed by triple antibiotic ointment. Mice were then placed into a 37°C incubation chamber for 20–30 min to recover. Mice were sacrificed at 3 days post-ischemia (dpi). Mice were euthanized by primary method of transcardial perfusion and secondary method of decapitation according to IACUC protocol #20190021. To determine how NRG-1 treatment affected the spatial proteomic profiles following MCAO, mice were randomly divided into two treatment groups and injected i.p. with either a daily 100 μL dose of vehicle (0.1% BSA in PBS; *n* = 4) or NRG-1 (5μg/kg/day NRG-1β (EGF-like domain, R&D Systems, Minneapolis, MN) in 1% BSA in PBS; *n* = 3) beginning 24 h after surgery, then sacrificed at 3 dpi. All NRG-1 and vehicle treatment studies were performed such that the surgeon was unaware of the treatment used before the procedure.

### Histology and immunohistochemistry

After MCAO studies, mice were euthanized by deep anesthesia with 5% isoflurane and perfused transcardially with saline followed by cold 4% paraformaldehyde (PFA) solution (Cat# HT501128, Sigma, Burlington, MA). Brains in preparation for histological and immunohistochemical analysis were quickly removed after transcardial perfusion and maintained in 4% PFA for 24 h before being cryoprotected in 30% sucrose/PBS (Sucrose: Cat# C12H22O11, Fisher Scientific, Hampton, NH; PBS: Cat# SH30258.02, HyClone, Logan, UT). The brains were then flash frozen and stored at −80°C until sectioning. Coronal sections of 12–15 μm thickness were cryosectioned and mounted on slides which were then stored at −80°C until further processed. Adjacent sections were used for correlating stains at each stereotaxic location.

Cresyl violet (Cat# C5042, Millipore, Billerica, MA) stain was first reconstituted from powder with distilled water, allowed to stir overnight, and then 0.3% glacial acetic acid (Cat# A38SI-212, Fisher Scientific, Hampton, NH) was added and mixed thoroughly. Sections were stained with cresyl violet on slides beginning with rehydrating steps of 15 min incubation with 95% ethanol (Cat# EX0276-4, Millipore, Billerica, MA), 1 min with 70% ethanol, 1 min with 50% ethanol, 2 min with distilled water, and 1 min with distilled water. Sections were then stained with cresyl violet warmed to 37.5°C for 3 min followed by distilled water for 1 min. Sections were then dehydrated with 1 min of 50% ethanol, 2 min of 70% ethanol with 1% glacial acetic acid, 2 min of 95% ethanol, and 1 min of 100% ethanol. After allowing the ethanol to dry off the sections, they were incubated with warmed cresyl violet again for 2 min followed by distilled water for 2 min. Sections were cleared with a 5-min wash of Histoclear (Cat# H2779-1L, Sigma, Burlington, MA) and mounted and cover slipped with DPX (Cat# 06522, Millipore, Billerica, MA).

Fluoro Jade B (FJB; Cat# AG310, Millipore, Billerica, MA) labeling was performed as we previously described, to ensure infarct presence and record infarct size and location (Noll et al., 2019). Sections were post-fixed with 10% formalin for 10 min and then washed twice with PBS for 5 min. Sections were then directly incubated in 0.06% potassium permanganate (KMnO_4_; Cat# 6360-16, Ricca Chemical, Arlington, TX) for 3 min followed by distilled water for 2 min. Sections were then incubated in a freshly prepared solution of 0.0004% FJB with DAPI (Cat# ab228549, Abcam, Waltham, MA) for 20 min, rinsed in distilled water 3 times for 2 min, and then dried at 50°C.

For immunohistochemical studies, sections were dried at room temperature for 30 min. After rinsing with 0.01M PBS, sections were blocked in PBS containing 5% normal donkey serum (Cat# ab7475, Abcam, Waltham, MA) and 0.1% Triton X-100 (Cat# X100, Sigma, Burlington, MA) for 1–2 h at room temperature, rinsed with PBS/0.2% Tween-20 (PBST; Cat# 01512, Chem-Impex International, Wood Dale, IL) and, then incubated overnight at 4°C with primary antibodies of polyclonal rabbit anti-Iba-1 (1:1000, Cat# 019-19741, Wako, Osaka, Japan) and Cy3-conjugated monoclonal mouse anti-GFAP (1:400, Cat# C9205, Sigma, Burlington, MA). Sections were washed 3 times with PBST, incubated with respective AlexaFluor 594-conjugated donkey anti-rabbit IgG antibody (1:400, Cat# 711-585-152, Jackson ImmunoResearch Laboratory, West Grove, PA) for 1 h at room temperature, then rinsed 4 times with PBST before mounting with DAPI-Fluoromount-G (Cat# OB010020, Fisher Scientific, Pittsburgh, PA).

### Immunohistochemical quantification

A Leica DM5500 B Automated Upright fluorescence microscope was used to capture all digital images of FJB stained sections at 5x magnification to capture the entire image area of FJB^+^ cells in the FITC channel at the same exposure time. Immunohistology images of FJB^+^ cells were captured at bregma +2 and +1. Images were taken at the ipsilateral core and corresponding contralateral cortical region of three separate tissue sections for each mouse (MCAO, *n* = 4 biological replicates; MCAO+NRG-1 *n* = 3 biological replicates; technical replicates= 3 for all). FJB^+^ cells were counted semi-automatically with ImageJ software (Media Cybernetics, Inc., Bethesda, MD) after threshold at size 10-infinity (pixel units) and circularity 0.4–1.00. FJB^+^ cell counts were averaged for each stereotaxic location.

A Nikon TS2-S-SM inverted fluorescence microscope equipped with a CCD camera was used to capture all digital images of Iba-1 and GFAP sections at 10x magnification in the TRITC channel. Iba-1 and GFAP images were captured at the same exposure times, respectively for each marker. Immunohistology images of Iba-1, and GFAP were captured at approximately bregma +2, +1, and 0 at four different regions of interest: the core, core border, peri-infarct normal tissue (PiNT) and contralateral cortex in three separate corresponding tissue sections for each mouse. Mean gray values were calculated with ImageJ2 1.53d (Fiji) software. All picture properties were obtained and ensured for consistency. Pictures were converted to 32-bit gray before analysis, and pictures were not altered in any other way. Area fraction was utilized as a control where all picture values must have 100% area fraction. Mean gray value region of interest (ROI) technical replicates were normalized against the averaged mean gray value of contralateral ROIs. Iba-1 maxima count was analyzed with prominence=15, in areas of high tissue damage (based on cresyl violet staining) where background signal may be higher, prominence was increased to 18. Maxima count ROI technical replicates were normalized against the averaged mean gray value of contralateral ROIs. Image collection and data analysis were performed by an individual who was blinded to the experimental conditions.

### Formalin-fixed paraffin embedded tissue preparation

Brains in preparation for Nanostring GeoMx DSP were continually dehydrated in preparation for paraffin embedding: 24 h of 4% PFA was followed by 24 h each in 40% ethanol, 70% ethanol, and a second change of 70% ethanol (MCAO, *n* = 3 biological replicates; MCAO+NRG-1, *n* = 4 biological replicates). Only male mice were used in these studies to decrease variability. Brains were then placed whole into cassettes and processed in a Core Facility Tissue-Tek Processor in a 12-h cycle before embedding coronally in paraffin. The 12-h cycle consisted of an initial 30-min ethanol wash, 5x 1 h ethanol washes, 3x 45-min incubation in CitriSolv, and 3x 1 h incubation in paraffin wax before embedding (all reagents for the Tissue-Tek Processor were provided by the core facility).

Formalin-fixed, paraffin-embedded (FFPE) samples were sectioned to approximately bregma +2 and 5 μm thickness sections were mounted onto slides and allowed to dry at room temperature overnight. FJB labeling was performed as described above to ensure infarct presence and record infarct size and location. Samples confirmed with FJB^+^ cells indicating successful MCAO were sent to Nanostring (Seattle, WA) for tissue preparation and GeoMx DSP profiling. The GeoMx DSP Protein Assay protocol is described in detail in the GeoMx DSP Manual Slide Preparation User Manual (MAN-10150-01, page 12–26).

### Nanostring digital spatial profiling analysis

To visualize whole tissue, mounted slides were stained with oligo-conjugated antibodies for MAP2, Iba-1 and GFAP, and with Syto13 (nuclei) in the GeoMx DSP instrument as we previously described (Noll et al., 2022). Circular geometric patterns (200 μm diameter) were used to identify six ROIs on the scanned tissues of each sample: core border (CoreB), peri-infarct (Peri), and PiNT regions on ipsilateral and contralateral hemispheres. The GeoMx Neural Cell Profiling protein panel (Table 1; 68 target proteins + 3 IgG background control proteins) was utilized for this analysis. UV-cleavable oligo-conjugated antibodies according to the panel were dispensed onto each ROI, UV-cleaved off, aspirated into a plate, hybridized and counted by the GeoMx DSP instrument. All resulting spatial, quantified analysis was performed in the Nanostring GeoMx Data Analysis Suite software (v2.2). Background correction was determined by protein target correlation plot and high correlation was seen between Rb IgG, Rb IgGa, and Rb IgGb. All three IgG proteins were used for panel background correction *via* signal-to-background ratio. For detailed information on protein analyte validation, see the Nanostring Whitepaper (MK2598): https://www.brukerspatialbiology.com/wp-content/uploads/WP_GeoMx_Antibody_Validation_White_Paper.pdf.

**Table 1:** Nanostring DSP neural cell panel target proteins.

Protein name	Gene	Full target name
Aldh1l1	Aldh1l1	Aldehyde dehydrogenase 1 family, member L1
Alpha-synuclein	Snca	Synuclein, alpha
Amyloid Precursor Protein	App	Amyloid beta (A4) precursor protein
Amyloid-Beta 1-42	App	Amyloid beta (A4) precursor protein
ApoA-I	Apoa1	Apolipoprotein A-I
APOE	Apoe	Apolipoprotein E
ATG12	Atg12	Autophagy related 12
ATG5	Atg5	Autophagy related 5
BACE1	Bace1	Beta-site APP cleaving enzyme 1
BAG3	Bag3	BCL2-associated athanogene 3
Beclin-1	Becn1	Beclin 1, autophagy related
Calbindin	Calb1	Calbindin 1
CD11b	Itgam	Integrin alpha M
CD163	Cd163	CD163 antigen
CD31	Pecam1	Platelet/endothelial cell adhesion molecule 1
CD39	Entpd1	Ectonucleoside triphosphate diphosphohydrolase 1
CD40	Cd40	CD40 antigen
CD45	Ptprc	Protein tyrosine phosphatase, receptor type, C
CD68	Cd68	CD68 antigen
CD9	Cd9	CD9 antigen
CSF1R	Csf1r	Colony stimulating factor 1 receptor
Ctsd	Ctsd	Cathepsin D
GAPDH	Gapdh	Glyceraldehyde-3-phosphate dehydrogenase
GFAP	Gfap	Glial fibrillary acidic protein
GPNMB	Gpnmb	Glycoprotein (transmembrane) nmb
Histone H3	H3c1	H3 clustered histone 1
IBA1	Aif1	Allograft inflammatory factor 1
IDE	Ide	Insulin degrading enzyme
ITGAX	Itgax	Integrin alpha X
Ki-67	Mki67	Antigen identified by monoclonal antibody Ki 67
LC3B	Map1lc3b	Microtubule-associated protein 1 light chain 3 beta
LRRK2	Lrrk2	Leucine-rich repeat kinase 2
MAP2	Map2	Microtubule-associated protein 2
Mertk	Mertk	MER proto-oncogene tyrosine kinase
MHC II	H2-Ab1, H2-Eb1	Histocompatibility 2, class II antigen E beta
MSR1	Msr1	Macrophage scavenger receptor 1
Myelin basic protein	Mbp	Myelin basic protein
Neprilysin	Mme	Membrane metallo endopeptidase
NeuN	Rbfox3	RNA binding protein, fox-1 homolog (C. elegans) 3
Neurofilament light	Nefl	Neurofilament, light polypeptide
NRGN	Nrgn	Neurogranin
Olig2	Olig2	Oligodendrocyte transcription factor 2
P2RX7	P2rx7	Purinergic receptor P2X, ligand-gated ion channel, 7
P62	Sqstm1	Sequestosome 1
Park5	Uchl1	Ubiquitin carboxy-terminal hydrolase L1
Park7	Park7	Parkinson's disease (autosomal recessive, early onset) 7
Phospho-Alpha-synuclein (S129)	Snca	Synuclein, alpha
Phospho-Tau (S199)	Mapt	Microtubule-associated protein tau
Phospho-Tau (S214)	Mapt	Microtubule-associated protein tau
Phospho-Tau (S396)	Mapt	Microtubule-associated protein tau
Phospho-Tau (S404)	Mapt	Microtubule-associated protein tau
Phospho-Tau (T231)	Mapt	Microtubule-associated protein tau
PINK1	Pink1	PTEN induced putative kinase 1
PLA2G6	Pla2g6	Phospholipase A2, group VI
PSEN1	Psen1	Presenilin 1
S100B	S100b	S100 protein, beta polypeptide, neural
S6	Rps6	Ribosomal protein S6
SPP1	Spp1	Secreted phosphoprotein 1
Synaptophysin	Syp	Synaptophysin
Tau	Mapt	Microtubule-associated protein tau
Tdp-43	Tardbp	TAR DNA binding protein
TFEB	Tfeb	Transcription factor EB
TMEM119	Tmem119	Transmembrane protein 119
Tyrosine Hydroxylase	Th	Tyrosine hydroxylase
Ubiquitin	Ubb	Ubiquitin B
ULK1	Ulk1	Unc-51 like kinase 1
Vimentin	Vim	Vimentin
VPS35	Vps35	VPS35 retromer complex component

### Pathway analysis

The protein datasets were analyzed using Ingenuity Pathway Analysis (IPA; Qiagen, Germantown, MD, USA; https://www.digitalinsights.qiagen.com) and overlaid onto a global molecular network developed from information contained in the QIAGEN Biomedical Knowledge Base. The canonical pathways that were most significant to the dataset were identified.

### Statistical analysis

Samples sizes were determined based on power calculations and findings from previous stroke studies performed at our laboratory (Noll et al., 2019, 2022). Averaged normalized mean gray values of ipsilateral ROIs from immunohistochemistry micrographs were compared against the averaged contralateral ROIs in correlating bregma locations. Significance of the results was tested with a multiple *t*-test based on their normality of distribution (Shapiro-Wilk test) using Graphpad Prism 9 (GraphPad Software, San Diego, CA). *P*-value < 0.05 was considered as significant. A Q-Q plot showed that the data were normally distributed (not shown). Gene expression analysis was performed using the GeoMx Data Analysis Suite with built-in statistical analysis (Nanostring Technologies, Seattle, WA) according to manufacturer's instructions. GeoMx DSP spatial ROIs were compared using a linear mixed model (LMM) with Benjamini-Hochberg FDR (BH) correction and random effect for Scan ID and ROI ID. Fold changes were identified by comparing the ROI/contralateral ROI with a significance of *p* value < 0.05. Fold change values represent log2-transformed fold change relative to contralateral ROI. Therefore: positive values indicate upregulation, negative values indicate downregulation, a value of −1 represents a 2-fold decrease and a value of +1 represents a 2-fold increase.

## Results

### Delayed NRG-1 treatment does not affect neuronal death or glial cell levels

**Figure 1:**
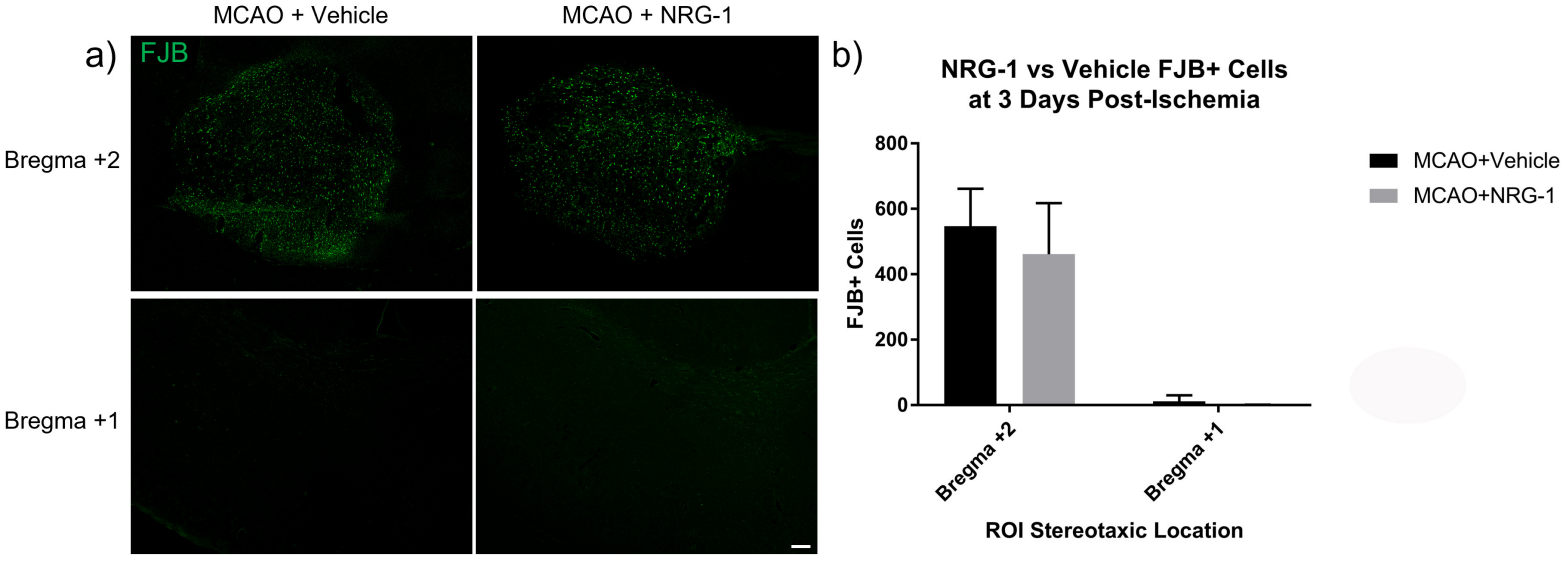
FJB demonstrates no difference with NRG-1 treatment at 3 days post-ischemia. Brains from mice subjected to MCAO and treated blindly with vehicle or NRG-1 were examined 3 days after ischemia. **(a)** Representative FJB-stained images of MCAO+Vehicle and MCAO+NRG-1 groups at bregma +2 and +1 (5 × magnification). **(b)** Quantification showed FJB+ cells throughout the infarct core at bregma +2, with no significant difference between groups at bregma +2. Data are expressed as mean ± SD (MCAO+Vehicle, *n* = 4; MCAO+NRG-1, *n* = 3; *p* < 0.05). Scale bar = 100 μm.

We examined the infarct, and neuroprotection in vehicle and delayed NRG-1 treated mice at 3 dpi using FJB. Animals were treated daily with NRG-1 or vehicle 24 h following MCAO. After MCAO and NRG-1 treatment, brain tissue was collected and examined spatially at 3 days after ischemia at stereotaxic locations bregma +2 and +1. FJB^+^ cells were found throughout the infarct core at bregma +2 with no difference in cell number between treatment groups (Figure 1). Vehicle group exhibited a mean of 547.3 ± 114 and NRG-1 treatment exhibited a mean of 461.4 ± 156 at bregma +2. Very few FJB^+^ cells were found at bregma +1 and no FJB^+^ cells were found at bregma +1 (not shown) in either treatment group.

**Figure 2:**
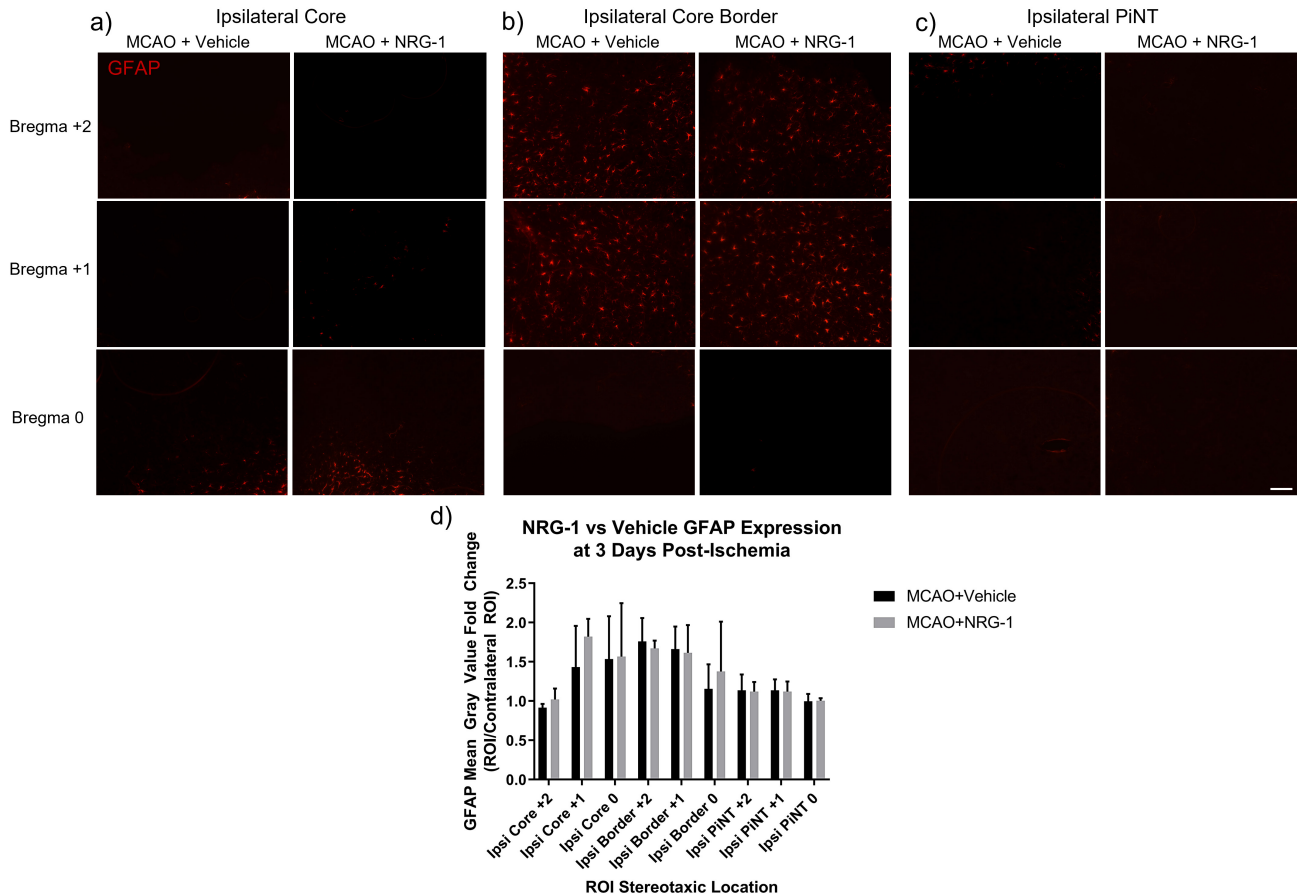
GFAP expression shows no difference with NRG-1 treatment at 3 days post-ischemia. Brains from MCAO mice treated with vehicle or NRG-1 were assessed for GFAP expression 3-days post-ischemia. **(a–c)** Mean gray value fold change was measured in ipsilateral core, core border, and peri-infarct (PiNT) regions relative to contralateral hemisphere. **(d)** No significant differences were observed between treatment groups at any location. Data are expressed as mean ± SD (MCAO+Vehicle, *n* = 4; MCAO+NRG-1, *n* = 3; *p* < 0.05). Scale bar = 100 μm.

**Figure 3:**
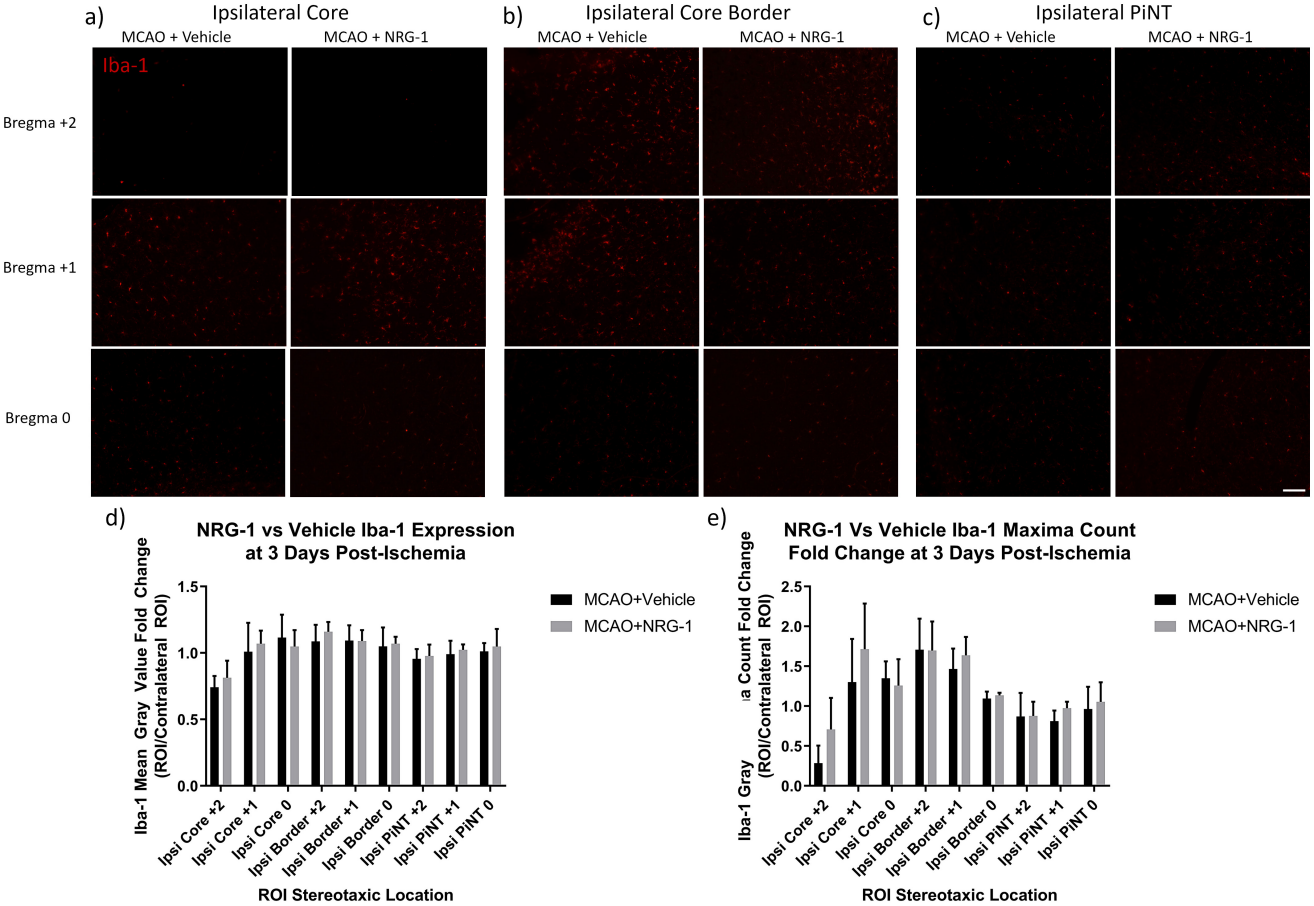
Iba-1 expression shows no difference with NRG-1 treatment at 3 days post-ischemia. Mice were treated with vehicle or NRG-1 after MCAO and analyzed for Iba-1 expression. **(a–c)** Mean gray value fold change in ipsilateral core, core border, and PiNT regions. **(d, e)** Data are expressed as mean ± SD (MCAO+Vehicle, *n* = 4; MCAO+NRG-1, *n* = 3; *p* < 0.05). Scale bar = 100 μm.

**A**strocyte and microglial levels were assessed in a spatial manner with the immunohistology markers, GFAP and Iba-1, respectively. Mice were blindly treated with vehicle or NRG-1 after MCAO induction and brain tissue was collected and examined spatially at 3 days after ischemia at stereotaxic locations bregma +2, +1, and 0 within the ipsilateral core, core border, and PiNT for GFAP and Iba-1 expression. GFAP expression was analyzed as mean gray value fold change against the contralateral side to detect regional changes in GFAP expression. GFAP regional fold change expression in MCAO animals was compared between treatment groups (Figure 2). No significant changes in GFAP mean gray value fold changes between MCAO+Vehicle and MCAO+NRG-1 treatment groups were seen in any region at any stereotaxic location. Iba-1 regional fold changes and gray maxima count fold changes in MCAO animals was compared between treatment groups (Figure 3). No significant changes in Iba-1 mean gray value fold changes or gray maxima count fold changes between MCAO+Vehicle and MCAO+NRG-1 treatment groups were seen in any region at any stereotaxic location. We acknowledge the relatively modest sample size, however statistical analyses were conducted appropriately and effects were consistent across biological replicates.

### NRG-1 treatment elucidates an early regenerative profile with nanostring DSP

**Figure 4:**
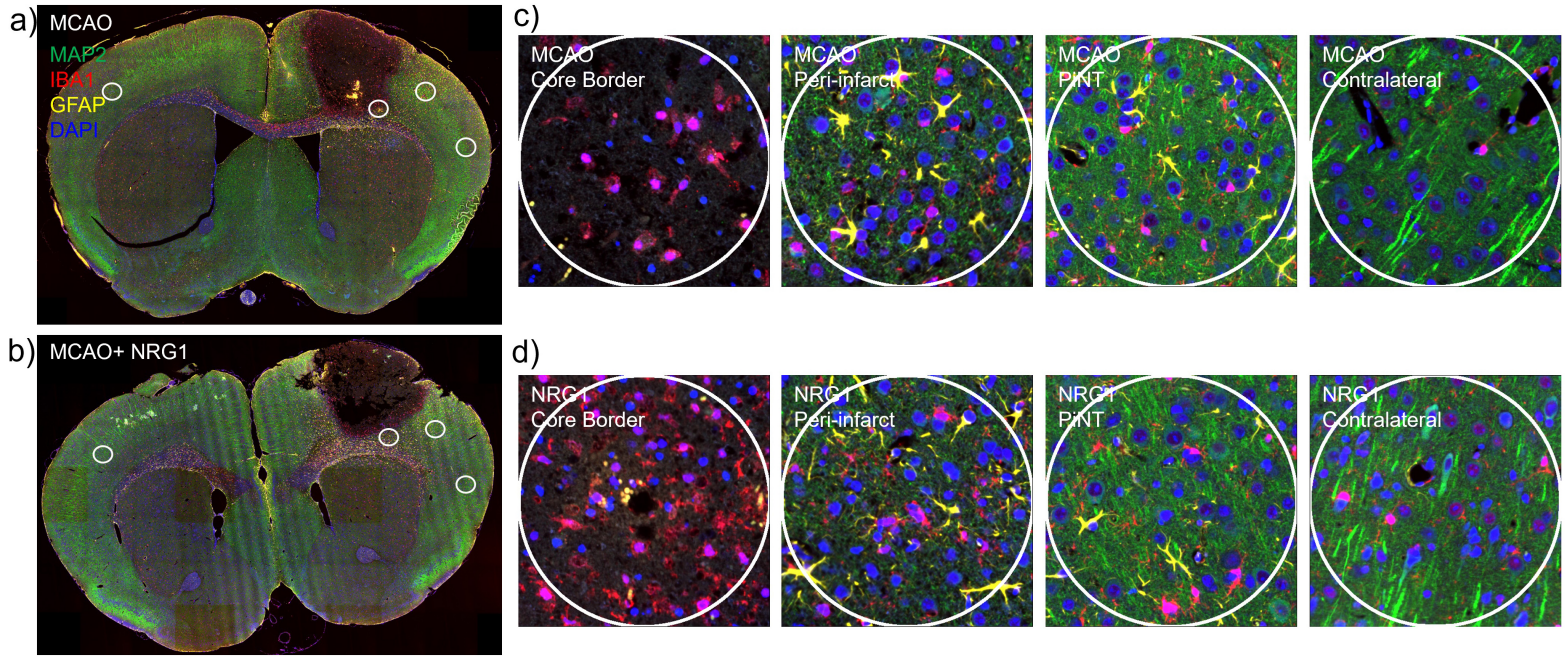
Representative immunohistochemical images for DSP analysis and ROI selection. Coronal sections from MCAO mice treated with vehicle **(a)** or NRG-1 **(b)** were immunostained for MAP2 (green), GFAP (yellow), Iba-1 (red), and Syto13 (blue). Overlay images illustrate ROI selection (ipsilateral core border, peri-infarct, PiNT, and contralateral cortex, outlined in white). **(c, d)** Higher magnification ROIs from MCAO and NRG-1 treated groups show similar morphology between conditions. (MCAO+Vehicle, *n* = 3; MCAO+NRG-1, *n* = 4). Scale bars: *a, b* = 1 mm; c, *d* = 20 μm.

We previously showed that a unique spatial protein profile was induced after ischemic stroke using Nanostring's GeoMx DSP (Noll et al., 2022). Here, we used DSP to elucidate if delayed NRG-1 treatment initiates a spatial proteomic effect after stroke. Brain tissue from mice with MCAO and treated with NRG-1 were examined 3 days after ischemia in the ipsilateral CoreB, Peri, and PiNT in contrast to the respective contralateral side with the Nanostring DSP Neural Cell Profiling protein panel. In our previous publication, we showed that only one protein significantly changed (GFAP) when comparing the contralateral to the ipsilateral sides of the brain using this same stroke model and protein panel. Therefore, we chose to use the contralateral side as control in these studies because the sample preparation is identical on both sides, thus reducing the chance for variability (Noll et al., 2022). Sections were immunostained with four chosen identifying markers for ROI selection (Figure 4). Markers included: MAP2 for neurons, GFAP for astrocytes, Iba-1 for microglia, and Syto13 for nuclei. ROIs were in CoreB, Peri, PiNT and contralateral control (CC) corresponding to CoreB. The full neural cell profiling protein panel was run on each tissue resulting in quantified protein read-outs for each ROI. Each ROI was examined and compared to the contralateral ROI.

**Figure 5:**
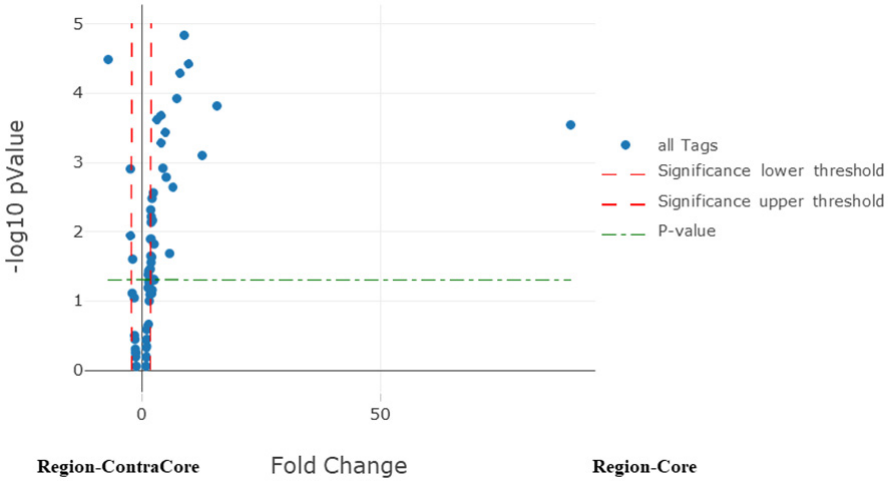
Ischemia produces region-specific proteomic profiles. Volcano plot showing transcript distribution by fold change (x-axis) and statistical significance (–log10 *p*-value, y-axis). Red dashed lines denote fold-change thresholds; green dashed line indicates *p*-value cutoff (*p* = 0.05). Significantly altered proteins are positioned outside threshold boundaries. (MCAO+Vehicle, *n* = 3; MCAO+NRG-1, *n* = 4).

To identify transcriptional differences between the CoreB and CC, differential expression analysis was performed and visualized using a volcano plot (Figure 5). The analysis revealed that most proteins clustered near the origin, indicating relatively small fold changes. However, several proteins exceeded both the fold-change thresholds (red dashed vertical lines) and the *p*-value cutoff (green dashed horizontal line), signifying statistically significant differential expression. Notably, a subset of proteins demonstrated robust upregulation in the Core relative to CC, while others were significantly downregulated, highlighting region-specific transcriptional responses.

**Figure 6:**
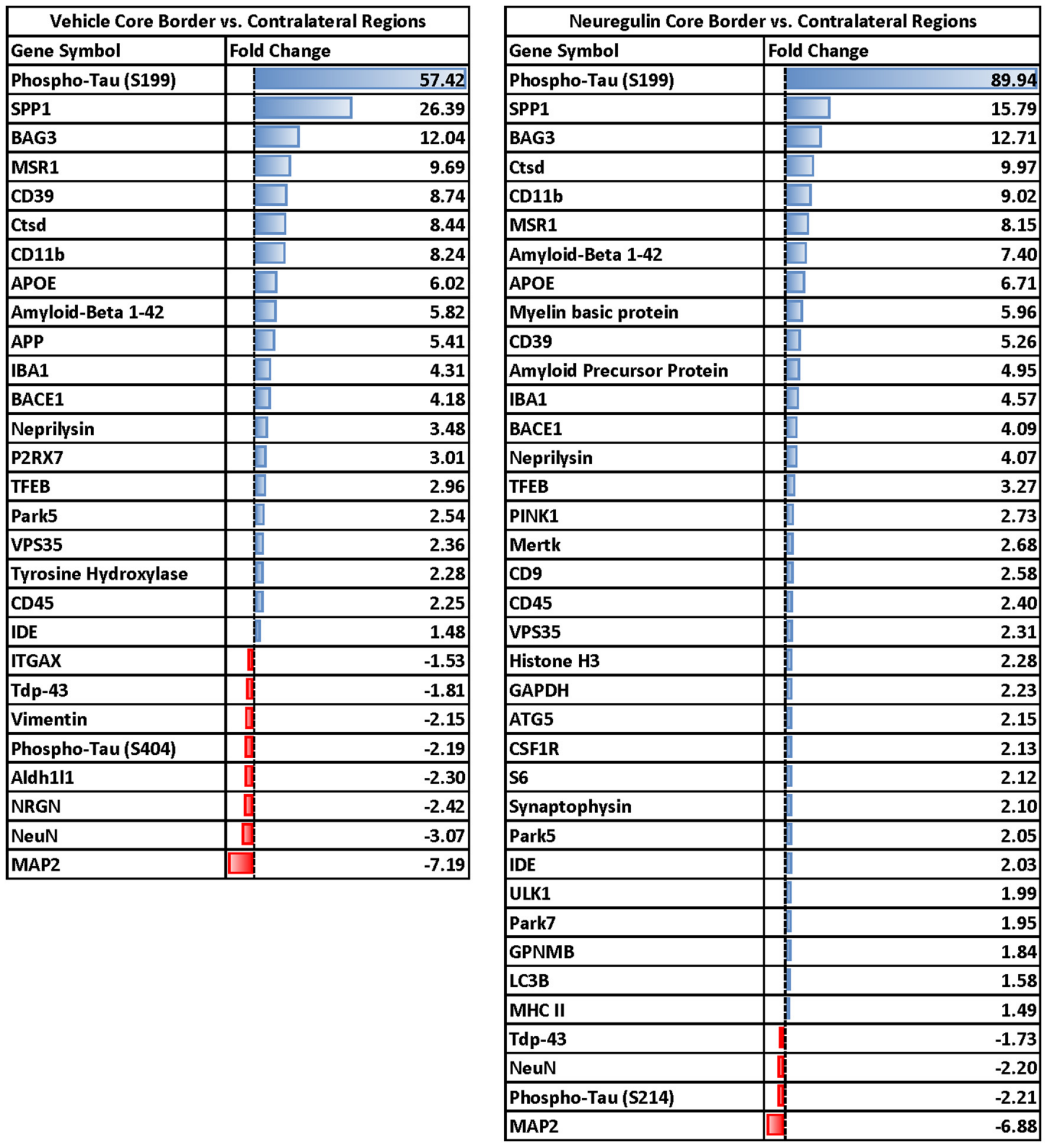
Comparative proteomic profiles in core regions. Vehicle-treated tissue showed enrichment of inflammatory and amyloid-related proteins, while NRG-1-treated tissue displayed elevated autophagy- and stress-response proteins, indicating distinct molecular responses to injury. (MCAO+Vehicle, *n* = 3; MCAO+NRG-1, *n* = 4).

Figure 6 presents comparative proteomic analyses of injured vs. contralateral tissue, stratified by treatment group (vehicle vs. neuregulin) and in the CoreB region. Proteins are listed with their corresponding fold-change values relative to contralateral tissue. Positive fold-changes indicate increased abundance, while negative fold-changes indicate reduced abundance. Ischemia in the CoreB region demonstrated 28 differentially regulated proteins, 20 upregulated and 8 downregulated, compared to the contralateral ROI (Figure 6A, Table 2). After ischemia and NRG-1 treatment, 33 proteins were upregulated and 4 were downregulated (Figure 6B, Table 2) in CoreB. NRG-1 treatment stimulated the upregulation of the synaptic outgrowth factor synaptophysin and myelin basic protein (MBP), an oligodendrocyte protein, which were not significantly altered by ischemia alone. SPP1, a marker of disease-related microglia was increased by 26.39-fold after MCAO and reduced to 15.79-fold with NRG-1 treatment. Ischemia also induced the expression of inflammatory proteins P2RX7 and CD39 which were reduced by NRG-1. NRG-1 treatment increased the levels of many proteins within the CoreB which were not altered by ischemia. Notably, neuregulin treatment was associated with induction of autophagy-related proteins (PINK1, ATG5, ULK1, and LC3B) and mitochondrial regulators (Park7, Mertk), suggesting engagement of clearance and repair mechanisms. NRG-1 treatment also demonstrated an increase in the CSF1 receptor, which has been shown to be upregulated after ischemia in microglia and neurons (Wang et al., 1999). When expressed on monocytes/macrophages it can also be associated with phagocytic capacity as well as proliferation (Jenkins et al., 2013). Phospho tau-S199 was upregulated by 57.42-fold in the core border by MCAO and further increased to 89.94-fold with NRG-1 treatment.

**Table 2:** Delayed NRG-1 treatment induces a late neuroprotective and early regenerative profile at 3 days post-ischemia.

		MCAO Core	NRG-1 Core	MCAO Peri-infarct	NRG-1 Peri-infarct	MCAO PiNT	NRG-1 PiNT
Protein name	Gene	Fold change	Fold change	Fold change	Fold change	Fold change	Fold change
Phospho-Tau (S199)	tau-S199	57.42	89.94	21.41	43.47		
Secreted phosphoprotein 1	SPP1	26.39	15.79	−24.59	−11.41		
Glial fibrillary acidic protein	GFAP			23.69	29.97	6.09	5.36
BAG family molecular chaperone regulator 3	BAG3	12.04	12.71	−4.79			
Macrophage scavenger receptor 1	MSR1 (cd204)	9.69	8.15	−6.13	−4.41		
Cluster of differentiation 39	CD39	8.74	5.26	−12.16	−5.95		
Cathepsin D	Ctsd	8.44	9.97	−6.90	−6.22		
Cluster of differentiation molecule 11B	CD11b	8.24	9.02	−7.92	−6.40		
Apolipoprotein E	ApoE	6.02	6.71	−10.61	−7.83		
Amyloid-Beta 1-42	αβ 1-42	5.82	7.40	−5.08	−4.11		
Myelin basic protein	MBP		5.96		−5.85		
Amyloid Precursor Protein	APP	5.41	4.95	−5.69	−3.83		
Ionized calcium binding adaptor molecule 1	IBA1	4.31	4.57	−3.11	−2.40		
Beta-secretase 1	BACE1	4.18	4.09	−4.79	−3.36		
Neprilysin	MME	3.48	4.07	−4.93	−3.25		
Transcription factor EB	TFEB	2.96	3.27	−2.81	−2.49		
P2X purinoceptor 7	P2RX7	3.01					
PTEN-induced kinase 1	PINK1		2.73				
MER proto-oncogene, tyrosine kinase	Mertk		2.68	−3.55			
Cluster of differentiation 9	CD9		2.58	−2.02	−2.01		
Parkinson's disease 5/UCHL-1	PARK5	2.54		−2.97	−2.18		
Marker of proliferation Ki-67	Ki-67				2.48		
Vacuolar protein sorting ortholog 35	VPS35	2.36			−1.82		
Tyrosine hydroxylase	TH	2.28		−3.21			
Cluster of differentiation 45	CD45	2.25	2.40		1.81		
Insulin-degrading enzyme	IDE	1.48	2.03	−1.77			
Histone H3	Histone H3		2.28				
Glyceraldehyde 3-phosphate dehydrogenase	GADPH		2.23				
Autophagy related 5	ATG5		2.15		−1.84		
Colony stimulating factor 1 receptor	CSF1R (CD115)		2.13		−1.77		
Ribosomal protein S6	S6		2.12				
Synaptophysin	SYP		2.10		−1.95		
Unc-51 like autophagy activating kinase 1	ULK1		1.99	−2.08	−1.88		
Parkinson's Disease 7	Park7		1.95	−2.19			
Glycoprotein non-metastatic melanoma protein B	GPNMB		1.84				
Microtubule-associated protein light chain 3B	LC3B		1.58				
Major histocompatibility complex II	MHC II		1.49		−1.69		
Microtubule associated protein 2	MAP2	−7.19	−6.88	6.32	6.57		
Neuronal nuclei/RNA binding fox-1 homolog 3	NeuN	−3.07	−2.20	2.77	2.29		
Neurogenin	NRGN	−2.42					
Aldehyde dehydrogenase 1 family member A1	Aldh1l1	−2.30		3.29	2.55		1.59
Phospho-Tau (S214)	tau-S214	−2.21			2.03		
Phospho-Tau (S404)	tau-S404	−2.19					
Vimentin	Vimentin	−2.15					
Tdp-43	TARDBP	−1.81	−1.73	1.94	1.70		
Integrin alpha X protein	ITGAX	−1.53					
CD88; macrosialin	CD68			−1.61			
Calbindin	Calb			−3.37	−3.25		
Apolipoprotein A-I	ApoA-I			−2.17			

Proteins with significant fold changes (p < 0.05, LMM) were identified in ipsilateral core border, peri-infarct, and PiNT regions compared to contralateral hemisphere. Proteins upregulated by ischemia and attenuated by NRG-1 are shown in green. Proteins unchanged following ischemia, but upregulated by NRG-1 are shown in yellow. MCAO+Vehicle (*n* = 3) and MCAO+NRG-1 (*n* = 4).

**Figure 7:**
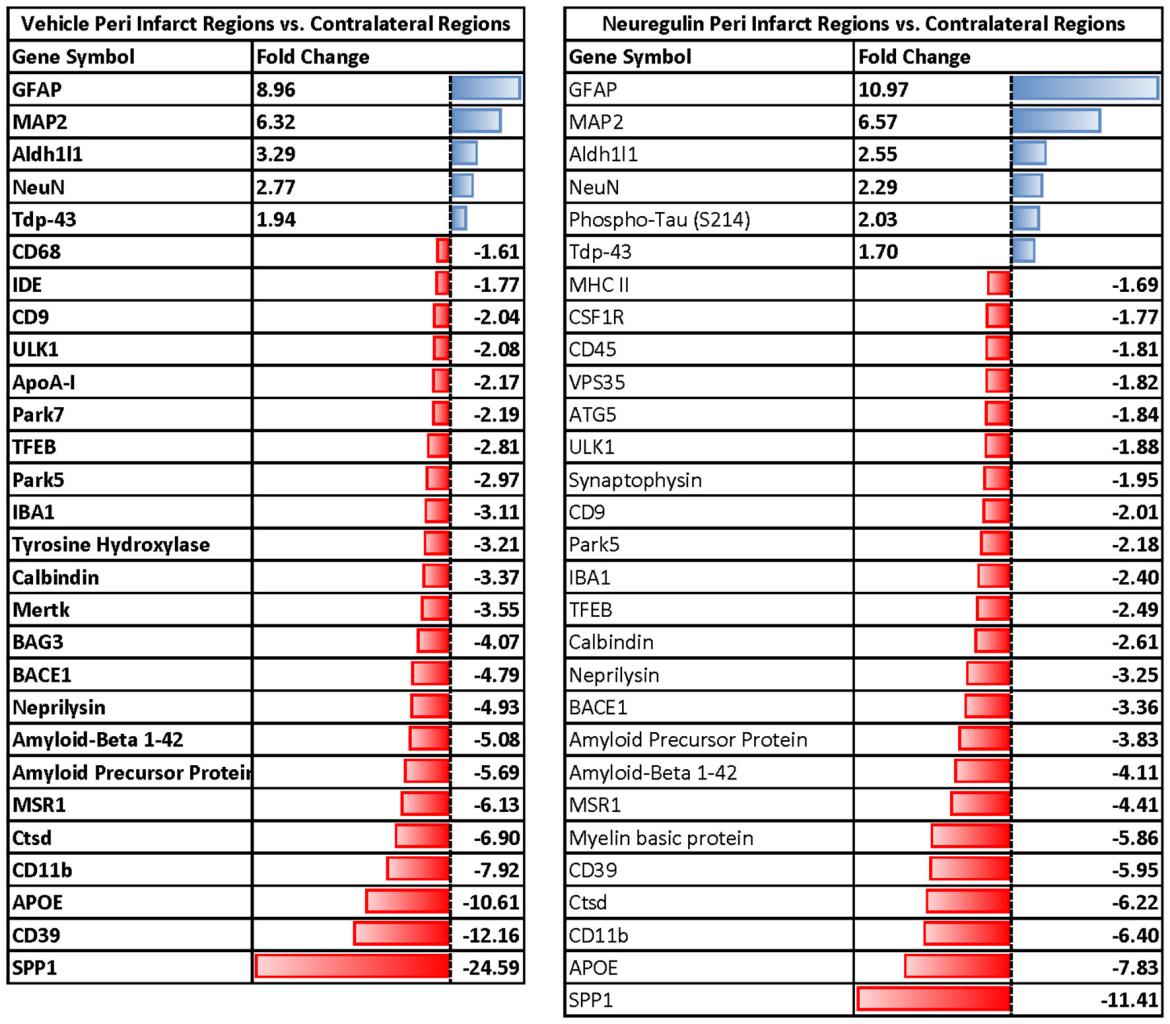
Comparative proteomic profiles in peri-infarct regions. Similar to core regions, vehicle-treated peri-infarct tissue showed enrichment of inflammatory and amyloid-related proteins, while NRG-1-treated tissue exhibited increased autophagy- and stress-response proteins. (MCAO+Vehicle, *n* = 3; MCAO+NRG-1, *n* = 4).

Ischemia in the Peri region resulted in 28 differentially regulated proteins, 5 upregulated and 23 downregulated (Figure 7A, Table 2). After ischemia and NRG-1 treatment, 6 proteins were upregulated and 23 were downregulated (Figure 7B, Table 2) in the Peri region. Vehicle-treated tissue exhibited marked increases in astrocytic (GFAP) and neuronal structural proteins (MAP2, NeuN), whereas NRG-1 treatment induced more modest neuronal marker expression but relative upregulation of stress-response proteins (Park7, TFEB, and ATG5). In both treatments, downregulated proteins included synaptic markers (Synaptophysin, PSD95), structural proteins (MAP2, Neurofilament), and neuronal identity markers (NeuN), indicating injury-associated neuronal loss or dysfunction. Several proteins were downregulated by ischemia, and the decrease was attenuated by NRG-1. These included SPP1, CD39, APOE, APP, BACE, BAG3, and MSR1. NRG-1 specifically increased phospho tau-S214, Ki-67, and CD45. which were not altered after MCAO.

### Pathway analysis

Pathway enrichment analysis was performed using IPA software on proteomic datasets to identify biological processes significantly altered following injury. The table lists the top canonical pathways for each comparison. For each pathway, the *p*-value indicates statistical significance, and the overlap column denotes the percentage and number of proteins detected relative to the total proteins in that pathway. The top five results of canonical pathways are shown (Table 3). In vehicle-treated samples, enriched pathways included amyloid fiber formation, neuroinflammation signaling, complement system activation, and neutrophil degranulation. By contrast, NRG-1 -treated tissues showed enrichment of pathways related to mitophagy, autophagy, and lysosomal biogenesis (CLEAR signaling). Both treatment conditions also showed overlap in amyloid- and cholesterol-related pathways (amyloid fiber formation, DHCR24 signaling).

**Table 3:** Canonical pathway enrichment in vehicle- and NRG-1–treated tissues.

Name	*p*-value	Overlap
Vehicle core borders vs. contralateral region		
Amyloid fiber formation	2.47E-05	4.1 % 3/73
Neuroinflammation signaling pathway	9.63E-05	1.3 % 4/317
DHCR24 signaling pathway	1.62E-04	2.2 % 3/137
Complement system	3.84E-04	5.4 % 2/37
Neutrophil degranulation	4.55E-04	0.8 % 4/476
Neuregulin core borders vs. contralateral region		
Mitophagy	2.26E-06	10.0 % 3/30
CLEAR signaling pathway	9.67E-05	1.4 % 4/285
Autophagy	8.43E-04	1.4 % 3/217
Macroautophagy	1.45E-03	3.1 % 2/65
Thyroid hormone biosynthesis	1.74E-03	50.0 % 1/2
Vehicle peri-infarct vs. contralateral region		
Amyloid fiber formation	4.41E-07	5.5 % 4/73
DHCR24 signaling pathway	5.49E-06	2.9 % 4/137
Post-translational protein phosphorylation	1.06E-04	2.8 % 3/107
Binding and uptake of ligands by scavenger receptors	1.21E-04	2.7 % 3/112
LXR/RXR activation	1.60E-04	2.4 % 3/123
Neuregulin peri-infarct vs. contralateral region		
Amyloid fiber formation	2.47E-05	4.1 % 3/73
DHCR24 signaling pathway	1.62E-04	2.2 % 3/137
Mitophagy	2.51E-04	6.7 % 2/30
Autophagy	6.22E-04	1.4 % 3/217
Amyloid processing	7.30E-04	3.9 % 2/51

Pathway analysis identified distinct biological processes: vehicle-treated tissue showed enrichment of inflammatory and immune-related pathways, whereas NRG-1–treated tissue was enriched in autophagy, mitophagy, and metabolic regulation pathways.

IPA network analysis was performed to determine the top molecular interaction networks associated with injury in different treatment groups and anatomical regions. The table lists major molecules included in each network, the IPA score (reflecting statistical significance of network enrichment), the number of focus molecules identified from the dataset, and the top diseases and functions associated with each network (Table 4). In vehicle-treated CoreB and Peri regions, networks were enriched for processes related to cellular maintenance, metabolic disease, immune activation, and inflammatory signaling. By contrast, neuregulin-treated regions showed networks incorporating autophagy- and mitophagy-related proteins (e.g., ATG5, ULK1, and PINK1), growth factor signaling (e.g., CSF1R, PDGF-BB, and VEGF), and stress response proteins (e.g., Hsp70, PARK7). Across conditions, common nodes included APOE, BACE1, MAP2, PI3K, and inflammatory mediators such as IL-1 and NFκB. The top associated functional categories included cellular compromise, neurological disease, metabolic pathways, and immunological responses, highlighting overlapping but distinct molecular signatures between treatment conditions.

**Table 4:** IPA networks in vehicle- and NRG-1–treated samples.

ID	Molecules in network	Score	Focus molecules	Top diseases and functions
IPA networks: vehicle core borders vs. contralateral regions				
1	Akt, ALDH1L1, Ap1, APOE, Aspartyl Protease, BACE1, calpain, Collagen type I (complex), Collagen(s), Creb, Fibrinogen, Gsk3, HDL, Hspg, IgG, IgM, IL1, IL12 (complex), IL12 (family), IgG, ITGAM, ITGAX, L-type calcium channel, LDL, LRP, MAP2, Mapk, MSR1, P2RX7, PKA, Pro-inflammatory Cytokine, SCAVENGER receptor CLASS A, TFEB, Tgf beta, VPS35	27	10	[Cellular function and maintenance, metabolic disease, organismal injury and abnormalities]
2	26S proteasome, APP, BAG3, Calmodulin, caspase, CD3, CK2, CTSD, cytokine, ENTPD1, ERK, ERK1/2, HDL/cholesterol, Hsp70, Hsp90 (family), IDE, Insulin, Jnk, MAP1LC3, Mek, NFkB (complex), Nrgn, P38 MAPK, PI3K (complex), Pkc(s), Proinsulin, PTPRC, Rbfox3, SGPP1, SRC (family), TARDBP, TCR, Tnf (family), Ubiquitin, Vegf	27	10	[Cellular assembly and organization, cellular function and maintenance, neurological disease]
3	APOBEC3G, CES1, CLEC12A, CLEC4C, CLEC4M, DRAP1, EIF1, EIF4EBP2, ENTPD1, GIMAP4, HERC6, Hrk, Ifi47, IFN type I receptor, IFNL2, ILRUN, Interferon alpha, IRF, LAIR1, LARGE1, LIFR, MARCHF2, MPL, MSR1, NKG7, NT5C3A, Oas, Oas1b, PARVG, RAD51AP1, RTP4, SARM1, SLFN13, SLFN5, Tgtp1/Tgtp2	4	2	[Antimicrobial response, infectious diseases, inflammatory response]
IPA networks: neuregulin core borders vs. contralateral regions				
1	Akt, APOE, ATG5, BACE1, C1Q (family), collagen type i (family), Creb, CSF1R, CTSD, Cyclin D, GAPDH, GPNMB, Gsk3, hemoglobin, Hsp70, IFN Beta, IgG, IL12 (complex), IL12 (family), Immunoglobulin, L-type calcium channel, MAP2, MERTK, MSR1, PARK7, PARP, PI3K (complex), PINK1, PKA, SCAVENGER receptor CLASS A, SPP1, TFEB, Tgf beta, ULK1, VPS35	48	16	[Cellular compromise, neurological disease, organismal injury and abnormalities]
2	26S proteasome, ALKBH1, AMPK, BAG3, CD3, CD9, CK2, cytokine, ENTPD1, ERK, ERK1/2, F Actin, FGFR, Histone h3, IDE, IL1, Insulin, Interferon alpha, Jnk, MAC, MAP1LC3, Mapk, Mek, NFkB (complex), P glycoprotein, P38 MAPK, Pkc(s), PLC gamma, PTK, PTPRC, selenomethionine, SRC (family), TCR, TMIGD2, Vegf	11	5	[Cellular compromise, organismal injury and abnormalities, skeletal and muscular disorders]
IPA networks: vehicle peri-infarct vs. contralateral regions				
1	Akt, ALDH1L1, Ap1, APOE, Aspartyl Protease, BACE1, calpain, Collagen type I (complex), Collagen(s), Creb, Fibrinogen, Gsk3, HDL, Hspg, IgG, IgM, IL1, IL12 (complex), IL12 (family), Immunoglobulin, ITGAM, ITGAX, L-type calcium channel, LDL, LRP, MAP2, Mapk, MSR1, P2RX7, PKA, Pro-inflammatory Cytokine, SCAVENGER receptor CLASS A, TFEB, Tgf beta, VPS35	27	10	[Cellular function and maintenance, metabolic disease, organismal injury and abnormalities]
2	26S proteasome, APP, BAG3, Calmodulin, caspase, CD3, CK2, CTSD, cytokine, ENTPD1, ERK, ERK1/2, HDL/cholesterol, Hsp70, Hsp90 (family), IDE, Insulin, Jnk, MAP1LC3, Mek, NFkB (complex), Nrgn, P38 MAPK, PI3K (complex), Pkc(s), Proinsulin, PTPRC, Rbfox3, SGPP1, SRC (family), TARDBP, TCR, Tnf (family), Ubiquitin, Vegf	27	10	[Cellular assembly and organization, cellular function and maintenance, neurological disease]
3	APOBEC3G, CES1, CLEC12A, CLEC4C, CLEC4M, DRAP1, EIF1, EIF4EBP2, ENTPD1, GIMAP4, HERC6, Hrk, Ifi47, IFN type I receptor, IFNL2, ILRUN, Interferon alpha, IRF, LAIR1, LARGE1, LIFR, MARCHF2, MPL, MSR1, NKG7, NT5C3A, Oas, Oas1b, PARVG, RAD51AP1, RTP4, SARM1, SLFN13, SLFN5, Tgtp1/Tgtp2	4	2	[Antimicrobial response, infectious diseases, inflammatory response]
IPA networks: neuregulin peri-infarct vs. contralateral regions				
1	Akt, ALDH1L1, Ap1, APH-1, APOE, Aspartyl Protease, BACE1, calpain, Collagen type I (complex), Collagen type IV, Collagen(s), Creb, CSF1R, Fibrinogen, Gsk3, HDL, Hsp27, Hsp70, IL1, L-type calcium channel, Laminin (complex), LDL, LRP, MAP2, Mmp, MSR1, PDGF-BB, PKA, Pkc(s), PLC, PP2A, Pro-inflammatory Cytokine, SCAVENGER receptor CLASS A, TFEB, Tgf beta	17	7	[Cellular assembly and organization, metabolic disease, organismal injury and abnormalities]
2	AMPK, ATG5, caspase, CD3, CD9, chemokine, CK2, cytochrome C, cytokine, ENTPD1, ERK, ERK1/2, IgG, IgM, IL12 (complex), IL12 (family), Immunoglobulin, Interferon alpha, ITGAM, Jnk, Mapk, Mek, NFkB (complex), P38 MAPK, PARP, PI3K (complex), PRKAA, PTK, PTPRC, Rac, SGPP1, SRC (family), TCR, Tnf (family), ULK1	17	7	[Cellular assembly and organization, cellular function and maintenance, immunological disease]
3	26S proteasome, APP, Aspartyl Protease, COA5, Csl, CTSD, CYP4V2, EEF1A, ELAPOR1, GFAP, GTDC1, HDL/cholesterol, Histone h3, Hspg, Insulin, MAP1LC3, miR-191-5p (and other miRNAs w/seed AACGGAA), Mt3, NNAT, NPFF, NPL, PDP2, pregnenolone sulfate, Rbfox3, SCN1A, selenomethionine, SPATA22, SPATA2L, TARDBP, TBC1D21, TMEM175, transglutaminase, Vegf, VPS35, ZNF302	14	6	[Cell morphology, developmental disorder, organismal injury and abnormalities]

Top IPA networks highlight divergent injury responses. Vehicle-treated tissue was enriched in pathways linked to inflammation, metabolism, and cellular stress, whereas NRG-1–treated tissue displayed networks enriched in autophagy, growth factor signaling, and stress-response pathways.

**Figure 8:**
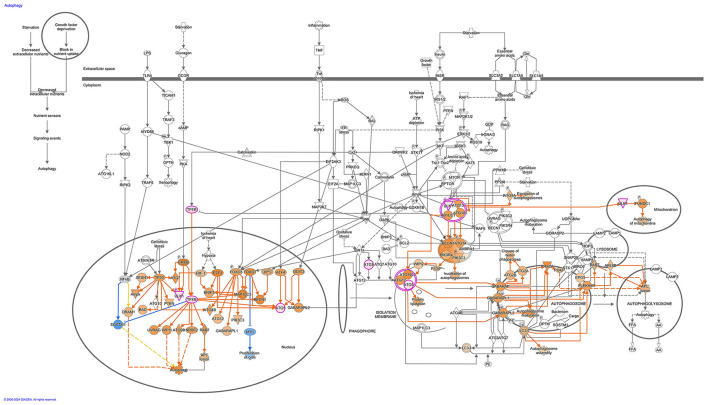
IPA autophagy pathway enrichment in NRG-1 treated core border tissue. Proteomic analysis revealed significant regulation of autophagy-related proteins, with pathway mapping demonstrating NRG-1–mediated enrichment of the autophagy canonical pathway.

Figure 8 depicts the autophagy canonical pathway generated by IPA. Pathway components highlighted in color indicate proteins identified as differentially regulated in the proteomic dataset. Orange nodes represent proteins with increased activity or predicted activation, while blue nodes indicate predicted inhibition. Pink outlines denote proteins present in the dataset, and solid or dashed lines indicate known direct or indirect molecular interactions, respectively. The analysis suggests that NRG-1 treatment is associated with multiple steps of the autophagy process, including initiation (ULK1 complex), nucleation (BECN1, ATG14), elongation and closure (ATG5–ATG12 conjugation system, LC3), and lysosomal fusion and degradation (LAMP proteins, autophagolysosome).

## Discussion

We previously showed that a unique spatial protein profile was induced after ischemic stroke using Nanostring's GeoMx Digital Spatial Profiling (DSP). Unlike bulk sequencing, which homogenizes samples and masks cellular diversity, spatial profiling maintains information on the precise localization of transcripts and proteins within their endogenous microenvironment. As we previously described, cerebral ischemia was characterized by increases in proteins associated with neuronal death, apoptosis, inflammation, and dysregulation of autophagy (Noll et al., 2022). Here our findings suggest that delayed daily NRG-1 modulates early spatial proteomic signatures associated with inflammation suppression and activation of regenerative and autophagy-related pathways following ischemic stroke. Our immunohistochemical results demonstrated no difference in neuronal cellular biomarkers between MCAO+Vehicle and MCAO+NRG-1 treatment groups, which was consistent with our DSP proteomic results. However, NRG-1 had a profound stimulating effect on the neuroregenerative outgrowth environment. Increased expression of MBP by NRG-1 could likely indicate proliferation and/or survival of oligodendrocytes as previous studies with NRG-1 treatment have shown, or presentation of the MBP antigen to promote an adaptive immune response (Alizadeh et al., 2017; Becker et al., 2011; Gauthier et al., 2013; Xu et al., 2012; Zierath et al., 2015). NRG-1 is recognized to act through its ErbB4 receptor, expressed on neurons and glia in the central nervous system (Buonanno and Fischbach, 2001). The ErbB4 receptor is enriched at the postsynaptic density and directly associates with PSD-95 as a role in synaptic transmission and neurite outgrowth (Buonanno and Fischbach, 2001; Huang et al., 2000; Gerecke et al., 2001). A study demonstrated that NRG-1 (GGF2/neuregulin 1β3) administered daily for ten days resulted in increased neural plasticity markers GAP-43 and synaptophysin in peri-infarct regions after stroke in rats (Iaci et al., 2016). Additionally, NRG-1 administered in a rat spinal cord injury model continuously for 1–2 weeks and showed enhanced axonal preservation, increased oligodendrogenesis, while reducing astrogliosis and neuronal degeneration (Gauthier et al., 2013).

Phosphorylated tau is increasingly recognized as an important player in secondary brain injury and long-term neurodegeneration after stroke, rather than the initial vascular event itself. After ischemic stroke, it has been shown that tau-proteins slowly hyperphosphorylate and accumulate and it has been suggested that the accumulation of phosphorylated tau-proteins contribute to the apoptotic process of neurons (Wen et al., 2004; Zheng et al., 2010). Experimental studies suggest that elevated S199 phosphorylation correlates with greater neuronal damage, infarct severity, and worse functional outcomes, and may also participate in triggering longer-term tau pathology that contributes to post-stroke cognitive decline. However, phospho-tau S199 upregulation has also been linked to neuroprotection, allowing neurons to resist apoptosis in relation to autophagy (Li et al., 2007b). Phospho-tau S199 increased 57-fold and 21-fold in the CoreB and Peri regions, respectively. NRG-1 treatment demonstrated a further significant increase in phosphorylated tau-S199 deposits in both the CoreB (90-fold) and Peri regions (43-fold) above the levels observed after stroke. An *in-vitro* study demonstrated that overexpression of phosphorylated tau at the site S199 can provide acute anti-apoptotic effects, but without clearance can lead to later neurodegeneration (Li et al., 2007b). This suggests that overexpression of tau-S199 in combination with enhanced autophagic flux with NRG-1 treatment may be associated with mechanisms of late neuroprotection for neurons, microglia, and potentially oligodendrocytes as well.

Pathway enrichment analysis findings suggest divergent biological responses to injury between vehicle and neuregulin treatment, with vehicle linked to inflammatory processes and NRG-1 linked to protein clearance and metabolic regulation. The results highlight autophagy as a key biological process associated with NRG-1 treatment in the CoreB region, supporting a role in cellular clearance and stress-response mechanisms. Autophagy is a critical process for maintaining cellular homeostasis *via* intracellular degradation (Klionsky et al., 2016). Autophagy serves as an adaptive response to the metabolic and oxidative stress triggered by cerebral ischemia. Autophagy plays a dual and context-dependent role in the pathophysiology of ischemic stroke, acting as both a neuroprotective and neurotoxic mechanism depending on its timing, intensity, and the cell type involved. In the acute phase of ischemia, moderate activation of autophagy serves as an adaptive response that supports neuronal survival under conditions of metabolic stress by recycling cytoplasmic components to sustain ATP levels. However, prolonged or excessive autophagy can shift from a protective to a destructive process, contributing to autophagic cell death and exacerbating neuronal injury (Adhami et al., 2007; Carloni et al., 2008; Levine et al., 2011; Mizushima and Komatsu, 2011). During ischemia, autophagic flux helps maintain energy homeostasis by recycling cytoplasmic macromolecules, thereby generating substrates for ATP production (Mizushima and Komatsu, 2011). In addition, autophagy limits the aggregation of misfolded proteins generated during ischemia and reperfusion (Carloni et al., 2008).

Mitochondrial integrity is a critical determinant of post-ischemic survival. Removal of depolarized mitochondria reduces reactive oxygen species (ROS) accumulation, prevents the release of pro-apoptotic proteins such as cytochrome c, and maintains ATP production (Klionsky et al., 2016; Sheng et al., 2010; Sheng and Qin, 2015; Zhang et al., 2013). This selective form of autophagy eliminates damaged mitochondria, preventing further oxidative stress and sustaining cellular bioenergetics. Nonetheless, overactivation of mitophagy can deplete functional mitochondria, aggravating energy failure and cell death.

Beyond metabolic regulation, autophagy intersects with inflammatory and apoptotic pathways. Cerebral ischemia triggers autophagy through several interconnected signaling pathways (Adhami et al., 2007; Wen et al., 2008). Oxygen–glucose deprivation (OGD) and ATP depletion activate AMP-activated protein kinase (AMPK), which phosphorylates and activates ULK1, initiating autophagosome formation. Concurrently, inhibition of mTOR removes its repressive effect on autophagy, facilitating LC3 lipidation and autophagosome maturation (Klionsky et al., 2016). Ischemic injury leads to mitochondrial depolarization and excessive reactive oxygen species (ROS) production, which in turn activate PINK1/Parkin-dependent mitophagy. Consistent with these mechanisms, ischemic brain tissue exhibits upregulation of canonical autophagy markers, including LC3-II, Beclin-1, ATG5, ATG7, PINK1, and Parkin, indicating enhanced autophagic and mitophagic activity. By degrading inflammasome components, autophagy suppresses NLRP3 activation and reduces IL-1β maturation, thereby attenuating neuroinflammation. In contrast, dysregulated or excessive autophagic flux during reperfusion can compromise lysosomal integrity, leading to cathepsin release and apoptotic amplification. Thus, precise temporal and molecular control of autophagy is essential to balance its protective and detrimental outcomes after ischemic stroke.

The PINK1/Parkin pathway is the most studied mechanism in stroke-related mitophagy. Upon mitochondrial depolarization, PINK1 accumulates on the outer mitochondrial membrane and recruits Parkin, which ubiquitinates mitochondrial proteins, targeting them for degradation (Zhao et al., 2018). In rodent models of MCAO, activation of PINK1/Parkin-dependent mitophagy reduced infarct volume and improved neurological outcomes. NRG-1 has been demonstrated to promote Parkin-dependent mitophagy in cardiomyocytes subjected to ischemia/reperfusion (I/R) injury. Postconditioning with NRG-1 increases mitophagic flux through a UCP2/PINK1/LC3B axis, thereby reducing infarct size and preserving myocardial function (Li et al., 2025). Taken together, this suggests that NRG-1 treatment may regulate autophagic and mitophagic flux after ischemia allowing for restoration for proper removal of degraded, misfolded, and damaged proteins and mitochondria in an antioxidant and neuroprotective manner.

Pharmacological modulators such as rapamycin, resveratrol, and metformin (autophagy activators) or 3-MA and chloroquine (autophagy inhibitors) have shown benefits in preclinical studies, but no therapy has yet translated into clinical practice (Galluzzi et al., 2017). NRG-1 treatment resulted in upregulation of many proteins related to autophagy and mitophagy in our DSP studies and it has been shown to regulate autophagy in various biological systems. NRG-1 mediated a decrease in cardiac ischemia/reperfusion injury *via* autophagy (Kundumani-Sridharan et al., 2021). In models of metabolic dysfunction–associated fatty liver disease (MAFLD), NRG-1 upregulated SIRT1, promoting autophagy, reducing steatosis, and limiting apoptosis (Xu et al., 2025).

We acknowledge that these findings are limited to a targeted protein panel. Therefore, the results should be interpreted as pathway-associated signatures rather than definitive pathway activation. Future studied using unbiased or functional assays will be required to confirm mechanistic pathways. Additionally, this study focused on early spatial proteomic changes at 3 days post-ischemia, rather than demonstrating functional recovery. Therefore, the functional implications of the observed molecular changes remain to be determined. However, previously published studies showed that NRG-1 administration 1–7 days after ischemic stroke in rats improved neurobehavioral outcomes (Iaci et al., 2010, 2016). Future studies will incorporate longitudinal behavioral assessments to determine whether delayed NRG-1 treatment confers sustained neurological recovery. We also recognize that these findings are consistent with engagement of autophagy-related pathways, rather than definitive evidence of increased flux. Future studies will incorporate LC3 turnover assays and p62 dynamics to directly assess flux.

In summary, our findings demonstrate that delayed NRG-1 treatment initiates a distinct spatial proteomic profile three days post-ischemia, characterized by reduced proinflammatory responses, enhanced neuronal plasticity and induction of autophagy and mitophagy in neuronal cells. Following ischemic stroke, NRG-1 can activate the AMPK pathway, as identified in the IPA network analysis, which restores autophagic flux and inhibits inflammatory signaling. Through binding to its receptor ErbB4, NRG-1 can trigger downstream cascades including PI3K/Akt, MAPK/ERK, and AMPK that promote autophagy and mitochondrial homeostasis. AMPK can initiate autophagy by activating ULK1 and upregulating LC3B, ATG5, and PINK1 (Zhao et al., 2018). Simultaneously, activated AMPK can inhibit the NLRP3 inflammasome by reducing mitochondrial ROS generation, enhancing mitophagy, and decreasing NF-κB-mediated transcription of inflammatory mediators. These coordinated actions can lead to reduced caspase-1 activation and IL-1β release, attenuating microglial and astrocytic inflammation. Collectively, NRG-1 may act as a dual modulator, coupling AMPK-dependent autophagy and mitochondrial repair with suppression of neuroinflammation, thereby promoting neuronal survival and recovery after ischemic injury. Table 5 summarizes the key molecular pathways affected by ischemia and restored by NRG-1 treatment.

**Table 5:** Key molecular pathways affected by ischemia and restored by NRG-1 treatment.

Pathway	Effect of ischemia alone	Effect of NRG-1 treatment
Neuroregeneration	↓ Neuronal markers	↑ MBP, synaptophysin
Inflammatory response	Exacerbated (microglial activation)	Attenuated (neuroprotective, anti-inflammatory niche)
NLRP3 inflammasome	Strongly activated → ↑ IL-1β, ↑ caspase-1	↓ NLRP3 activation, ↓ IL-1β, ↓ pyroptosis
Autophagy flux	Impaired	Restored via ULK1, LC3B, ATG5 upregulation
AMPKα signaling	Suppressed or dysregulated	↑ Phospho-AMPKα → Restores energy homeostasis

Despite over 100 clinical trials investigating neuroprotective compounds such as glutamate antagonists, anti-inflammatory agents, and free radical scavengers, none have demonstrated efficacy in stroke patients (Kidwell et al., 2001; O'Collins et al., 2006). This underscores the critical need for a multimodal therapeutic with the potential to extend the treatment window after ischemia. Encouragingly, clinical trials of NRG-1 in patients with congestive heart failure have demonstrated improved cardiac function, with safe and tolerable dosing (Lenihan et al., 2016; Parry et al., 2017). These findings provide strong translational support for advancing NRG-1 into clinical stroke studies for neuroprotection and neuroregeneration. Taken together, our study highlights NRG-1 as a promising candidate for post-ischemic intervention, offering both sustained neuroprotection and early neuroregeneration. Further investigation of NRG-1 in spatiotemporal detail across the phases of stroke recovery will be critical to fully realize its therapeutic potential.

## Data Availability

The datasets presented in this study can be found in online repositories. The names of the repository/repositories and accession number(s) can be found in the article/supplementary material.
